# EV-3, an endogenous human erythropoietin isoform with distinct functional relevance

**DOI:** 10.1038/s41598-017-03167-0

**Published:** 2017-06-16

**Authors:** Christel Bonnas, Liane Wüstefeld, Daniela Winkler, Romy Kronstein-Wiedemann, Ekrem Dere, Katja Specht, Melanie Boxberg, Torsten Tonn, Hannelore Ehrenreich, Herbert Stadler, Inge Sillaber

**Affiliations:** 1 Epomedics GmbH, Göttingen, Germany; 2 Clinical Neuroscience, Max Planck Institute of Experimental Medicine and DFG Research Center for Nanoscale Microscopy and Molecular Physiology of the Brain (CNMPB), Göttingen, Germany; 3 German Red Cross Blood Donor Service North-East, Institute of Transfusion Medicine, Dresden, Germany; 4 Institute of Pathology, Technische Universität München, Munich, Germany; 5 Department of Experimental Transfusion Medicine, Medical Faculty Carl Gustav Carus, Technische Universität Desden, Dresden, Germany

## Abstract

Generation of multiple mRNAs by alternative splicing is well known in the group of cytokines and has recently been reported for the human erythropoietin (EPO) gene. Here, we focus on the alternatively spliced *EPO* transcript characterized by deletion of exon 3 (hEPOΔ3). We show co-regulation of *EPO* and hEPOΔ3 in human diseased tissue. The expression of hEPOΔ3 in various human samples was low under normal conditions, and distinctly increased in pathological states. Concomitant up-regulation of hEPOΔ3 and *EPO* in response to hypoxic conditions was also observed in HepG2 cell cultures. Using LC-ESI-MS/MS, we provide first evidence for the existence of hEPOΔ3 derived protein EV-3 in human serum from healthy donors. Contrary to EPO, recombinant EV-3 did not promote early erythroid progenitors in cultures of human CD34+ haematopoietic stem cells. Repeated intraperitoneal administration of EV-3 in mice did not affect the haematocrit. Similar to EPO, EV-3 acted anti-apoptotic in rat hippocampal neurons exposed to oxygen-glucose deprivation. Employing the touch-screen paradigm of long-term visual discrimination learning, we obtained first *in vivo* evidence of beneficial effects of EV-3 on cognition. This is the first report on the presence of a naturally occurring EPO protein isoform in human serum sharing non-erythropoietic functions with EPO.

## Introduction

The cytokine erythropoietin (EPO) was firstly identified as the haematopoietic factor responsible for the proliferation and maturation of erythroid progenitor cells by preventing their apoptosis^1^. Meanwhile, EPO has been described as a protein with multifaceted properties. Besides stimulating erythropoiesis, EPO acts as growth and survival factor^1^ and is part of an endogenous tissue protection system^2–5^. Neuroprotective actions of EPO have been extensively shown *in vitro* (e.g. 6–8) and *in vivo* (e.g. 9–11) and efficacy in clinical trials using EPO for the treatment of a variety of degenerative disorders has been reported (e.g. 12–16).

Although the long scientific history of EPO – it was isolated in 1977^17^ from human urine and cloning of its gene was only several years later^18^ – alternatively spliced *EPO* transcripts were only recently described^19^: An *EPO* transcript variant with complete loss of exon 3 (denoted hS3) and a second variant with a partial loss of exon 4 (denoted hS4) were detected in samples of human kidney and foetal brain. The generation of multiple mRNAs by alternative splicing is well known in the group of cytokines, for example for IL-2^20^, IL-4^21^, IL-10^22^, IL-12^23^ and G-CSF^24^. Several splice variants are also known for *EPOR* ^25^. While some splice variants act as competitive inhibitors of the full-length protein^20, 21^, other acquire different biological functions^26^. For the recombinant protein products derived from the two alternatively spliced transcripts of *EPO*, hS3 and hS4 respectively, neuroprotective properties and in parallel, a lack of erythropoietic function were described^19^.

In the present study, we focussed on the splice variant hS3 (herein denoted as hEPOΔ3) and extended the panel of human samples for the detection of the spliced transcript according to the reported *EPO* expression^27–30^. Elevated levels of EPO were found in patients with chronic liver disease^31, 32^ and in individual samples of clear cell renal cell carcinoma (ccRCC)^33–35^. In order to test whether the splice variant hEPOΔ3 is also regulated under those pathological conditions, we established a realtime-PCR assay to quantify *EPO* and hEPOΔ3 transcript levels in human diseased (liver cirrhosis, ccRCC) and normal tissues. The primary factor for up-regulation of EPO expression is hypoxia (for review see 36) and the human hepatocellular carcinoma cell line HepG2 is known to express EPO^37^. Therefore, we used HepG2 cells to study hypoxia-related regulation of hEPOΔ3 compared to *EPO* in a model system.

Not all splice variants are functionally relevant and are often products of errors of the splicing process^38^. We expected a functionally relevant spliced *EPO* transcript to be translated into protein and secreted into the blood circulation. EPO itself is only present in picomolar concentrations in blood^39^. Further, the results obtained from the experiments mentioned above pointed to a low expression of the splice variant hEPOΔ3 under basal conditions. Therefore, in order to be able to detect the anticipated low levels of hEPOΔ3 derived protein (EV-3) and allow clear separation from EPO in serum of healthy human volunteers, we applied a multistep processing protocol and analysed the samples by LC-ESI/MS-MS. Based on the cDNA sequence of hEPOΔ3^19^ the corresponding protein EV-3 is supposed to lack amino acids encoded by exon 3. The peptide sequence spanning the last three amino acids encoded by exon 2 and the first six amino acids encoded by exon 4 is uniquely contained in EV-3 and therefore, allows its identification as well as differentiation to EPO in human serum samples.

For functional studies, EV-3 was produced by transient expression in CHO cells, the commonly employed cell line for production of recombinant EPO. Orienting our studies with EV-3 on previously described functional activity of EPO, we here focused on erythropoietic, neuroprotective and potential cognitive effects of the recombinant produced EV-3 in comparison to EPO. The results indicate that neuroprotective and beneficial effects on cognition are preserved in EV-3, whereas erythropoietic effects of EPO are not mediated by the EV-3 protein. Together with the observed co-regulation of the transcripts of EPO and EV-3 in human specimen we speculate that tissue protective and/or regenerative effects are part of the biological function of the here described EV-3 protein detected in the human serum.

## Methods

### PCR

Total RNA from human tissues (adult kidney, foetal brain) was purchased from Biocat (Germany). Total RNA from HepG2 cells was extracted using peqGold RNAPure (VWR, Germany) according to standard protocols. The Access-RT-PCR system from Promega (Germany) was used for reverse transcription and PCR amplification of *EPO* transcripts using RNA (3 µg) and *EPO*-specific forward and reverse primers (FWD: 5′-GATGGGGGTGCACGAAT GTCCTGC-3′; REV: 5′-CACACCTGGTCATCTGTCCCCTGTC-3′) according to manufacturer’s instructions. This PCR product was used for a second PCR amplification round using internal *EPO* primers (FWD: 5′-TCCTGTCCCTGCTGTCGCTC-3′; REV: 5′-CTGCAGGCCTCCCCTGTGTA-3′) and Pfu polymerase (Promega). PCR products were separated on agarose gels, purified using the QIAquick Gel Extraction Kit (Qiagen, Germany) and subcloned into the Zero Blunt TOPO vector (Thermo Fisher Scientific, Germany) for sequencing.

### Quantitative PCR analysis in human tissues

Human tissue samples were handled in accordance with the ethical rules of the Klinikum Rechts der Isar, Technical University Munich, for research use of surplus diagnostic materials. Written informed consent was obtained from all patients. Total RNA from formalin fixed paraffin-embedded (FFPE) human tissues was isolated using the High Pure RNA Paraffin Kit from Roche. In brief, tumour cells from clear cell renal cell carcinoma (ccRCC; n = 12) samples (Fuhrmann-grading G2 and G3) were macrodissected and compared to normal adjacent renal tissue. Cirrhotic liver tissues were compared to normal liver samples (n = 10). Total RNA from fresh human tissues (adult kidney, foetal brain, adult brain, liver and cirrhotic liver) was purchased from Biocat (Germany) and Amsbio (UK). RNA reverse transcription was performed on RNA (2 µg) with Superscript II, RNase Out, random primers and dNTPs. Primers specific for *EPO* transcript (SPEC_EPO; FWD: 5′-GTGCTGAACACTGCAGCTTG-3′; REV: 5′-CAGACTTCTACGGCCTGCTG-3′) and specific for hEPOΔ3 transcript (SPEC_EV-3; FWD: 5′-CACCACGCCTCATCTGTGAC-3′; REV: 5′-CTGCTGCCCGACCGTGA-3′) were ordered from Eurofins MWG Synthesis GmbH (Germany). QuantiTect primer assays for *Alas1* (Hs_Alas1_1_SG), *SRSF4* (Hs_SRSF4_1_SG) and *UBC* (Hs_UBC_1_SG) were purchased from Qiagen. Real-time PCR reactions were performed in the Lightcycler 480 apparatus using Light Cycler 480 SYBR Green I Master (Roche) with the following cycling: 5 min at 95 °C; 45 cycles: 10 sec at 95 °C, 17 sec at 55 °C, 10 sec at 72 °C; acquisition at 55 °C. SPEC_EV3 acquisition was done at 82 °C to avoid primer dimer generation, which significantly affects low copy EV-3 detection. Melting curve analysis was done to check for unspecific products. Efficiency of SPEC_EPO and SPEC_EV-3 assays was tested using dilution series of cDNA. Serial dilutions of plasmids containing *EPO* full-length and hEPOΔ3 inserts were used to test for primer specificity.

### *In silico* homology modelling

Homology modelling of EV-3 was performed at the SWISS-MODEL Protein Modelling Server database^40–43^ accessible via ‘http://swissmodel.expasy.org/’ using the EPO structure 1cn4.1.C.pdb as template.

### Recombinant proteins

Recombinant EV-3 and EPO proteins were produced by IBA GmbH (Germany) as Twin-Strep-Tag fusion proteins via transient transfection in CHO cells. Proteins were purified from cell culture supernatants using *Strep*-Tactin® Superflow® high capacity, dialyzed against D-PBS and sterile filtered. Silver staining of recombinant proteins after separation by SDS-PAGE revealed purity of >95%. The endotoxin contamination ranged from 8.3–15.1 EU/mg protein (Limulus Amebocyte Lysate Test, Endosafe nexgen PTS, Charles River), resulting in an endotoxin level of <0.01 ng/ml injection solution used for *in vivo* experiments.

### HepG2 culture and hypoxia

HepG2 cells were maintained in complete high-glucose DMEM medium (DMEM supplemented with 10% FCS and 1% penicillin/streptomycin). To study effects of hypoxia on *EPO* mRNA expression, cells were cultured in 6-well plates (1 × 10^6^ cells/well). After 24 hours, medium was exchanged and cultures were exposed to hypoxia (oxygen concentration <1%) or normoxia for 4 hours. Immediately thereafter, cells were lysed and RNA was extracted (miRNeasy Kit, Qiagen) and DNase digested. Reverse transcription was performed on RNA (1 µg) with Superscript III, RNase Out, random hexamers, oligo-dT primers and dNTPs. PPIA (Hs_PPIA_1_SG) primer assay was purchased from Qiagen. Real-time PCR reactions were performed in the Lightcycler 480 apparatus using the Power SYBR Green PCR Master Mix from Thermo Fisher Scientific with the following cycling: 2 min at 50 °C, 10 min at 95 °C; 45 cycles: 15 sec at 95 °C, 1 min at 60 °C, acquisition at 60 °C. Melting curve analysis was done to check for unspecific products. Primer dimer generation for SPEC_EV-3 was not detected in HepG2 samples.

### Analysis of HepG2 cell culture supernatants

Cell culture supernatants were harvested after 3–4 days, pooled and concentrated (Vivaspin, MWCO 10,000; Sartorius, Germany). For immunoprecipitation, biotinylated anti-EPO antibody (0.5 µg; BAF959, R&D) was added to concentrated supernatant (750 µl) and incubated over-night at 4 °C on a rotating shaker. Streptavidin-agarose was added and incubated for 1 hr at room-temperature. After three PBS-washing and centrifugation (700 g, 1 min, 4 °C) steps, agarose was re-suspended in SDS sample buffer and heated for 5 min at 99 °C. For subsequent analysis in mass spectrometry, proteins were eluted using 0.1 M glycine (pH 2.6) and neutralized with 2 M Tris (pH 8.5). Western Blotting was performed using a polyclonal rhEPO-antibody (H-162, Santa-Cruz) according to standard protocols; the ImageJ-program was used for densitometry analysis. Wheat germ affinity (WGA) chromatography was performed according to manufacturer’s instructions (Thermo Fisher Scientific) on concentrated HepG2 culture supernatants. Eluates from immunoprecipitation and WGA purification were desalted, concentrated and transferred to PNGase F (New, England Biolabs, Frankfurt, Germany) and endoprotease digestion (Asp-N/Glu-C) for analysis using multiple reaction monitoring (see below).

### Serum fractionation

Sera (n = 2; 20 ml of serum 1 and 100 ml of serum 2) of healthy human donors were fractionated via size exclusion chromatography. To this end, Superdex 75 columns for high resolution separation of biomolecules in the range of 3–70 kDa with different loading volume were used (XK 26/60: 2.6 cm diameter, 318 ml Superdex 75, flow rate set to 2.5 ml/min; BGP 100/950: 10 cm diameter, 5.2 L Superdex 75, flow rate set to 20 ml/min; GE Healthcare Life Sciences). The elution time of EPO was predicted from a calibration run (see Supplementary Figs S3a and S4a) using BSA (66 kDa) or a solution containing BSA and Azurin (30 kDa). Serum 1 (20 ml) was loaded on the XK 26/60 column and fractions of interest, having same elution times as Azurin in the calibration run, were pooled. The pool was concentrated using VivaFlow 50 (MWCO 10,000; Sartorius, Germany) and re-run twice on the XK 26/60 column until obtaining a narrow peak for serum albumin (Supplementary Fig. S3b). Serum 2 (100 ml) was first loaded on the BPG 100/950 column. Eluate fractions of interest according to the calibration run following the albumin peak, were pooled, concentrated and re-applied to the column. Elution fractions of the second run were chromatographed twice on the XK 26/60 column until obtaining a clear separation of proteins smaller than albumin (see Supplementary Fig. S4b). Final eluate fractions of serum 1 (C3 – C12, D2) and serum 2 (C4– C12, D1) were analysed separately. Using a commercial ELISA (Roche Diagnostics, Germany), EPO-positive fractions were identified and further purified using WGA chromatography. Eluates were PNGase F and endoprotease digested (Asp-N/Glu-C) for analysis using multiple reaction monitoring (see below).

### Multiple reaction monitoring (nano-LC-ESI-MS/MS)

Through a theoretical enzymatic digest peptides were searched which allow differentiation of EPO and EV-3 within one sample. Processing of the samples included digestion with PNGase F which not only removes N-linked oligosaccharides from glycoproteins but also deaminates glycosylated N-residues to D-residues (N → D). The subsequent cleavage of de-glycosylated proteins with endoproteases Asp-N (cuts before D) and Glu-C (cuts after E) generated EV-3 and EPO specific peptides: ‘DITVGQQAVE’ is specific for EV-3 and ‘DITTGCAE’ is specific for EPO (Table 1).

**Table 1:** Specificity of EV-3 and EPO “identifier” peptides.

Peptide	Protein containing peptide (UniProt Seq. ID)	Comment
DITVGQQAVE	no protein with 100% identity	specific for EV-3, detected by analysis
DITTGCAE	human erythropoietin (P01588)	specific for EPO, detected by analysis

Proteolytic peptides specific for EV-3 and EPO were predicted from the amino acid sequence and used for protein identification in multiple-reaction-monitoring (nano-LC-ESI-MS/MS). Blast is used to predict for possible homologies of these peptides to other proteins.

10 μg of PNGase treated sample was reduced with Dithiothreitol (DTT), alkylated with iodacetamide (IAA), then first digested with Asp-N (0.2 μg) at 37 °C over-night, then with Glu-C (0.2 μg) at room temperature for 4 hours. After completion of digest, the sample was lyophilized, re-dissolved in 0.1% formic acid (100 μl) and spiked with each isotopically labelled peptide (DITVGQQAVE; DITTGCAE). For nano-LC-ESI-MS/MS, total digested protein (3.5 μg) was applied. HPLC-separation was done on an Ultimate 3000 system (Dionex): Peptides were loaded onto a C18-column and subsequently separated on a PepMap™ analytical column (LC PACKINGS Nano SeriesTM PepMapTM C18) with linear gradients. Mass spectrometry was done using a QTRAP 5500 mass-spectrometer (ABSciex) coupled online to the HPLC-system. Identification and quantitation of the endogenous peptides, the EV-3 specific DITVGQQAVE and the EPO specific DITTGCAE, was based on retention times of the isotopically labelled peptides spiked to the enzymatically digested samples. Quantitation utilizes 5 transitions for each peptide. Standard curves were generated with linear regression as duplicates for each transition separately using peptide concentrations of 0.001, 0.01, 0.1, 1 and 10 fmol/μl.

### Colony forming unit (CFU) assay with human CD34+ cells

CD34+ cells were used in accordance with the guidelines approved by the Ethics Committee of the Dresden University of Technology. The informed consent was obtained from all donors. Granulocyte colony-stimulating factor (G-CSF) mobilized CD34+ cells from peripheral blood of two healthy donors were isolated by centrifugation over Biocoll (Biochrom) and by the use of CD34 microbeads and LS-MACS columns (Miltenyi Biotec; 94%  ±  3% purity). CD34+ stem cells, for each donor separately, were seeded in methylcellulose plates (500 cells per well, 6 well plates, Stem Cell Technologies, Canada) with 1.1 ml of medium (Methocult H4035 without EPO; Stem Cell Technologies). For treatment, different concentrations (0, 0.3, 3, 10, or 30 U/ml or weight equivalent) of rEPO or rEV-3 were supplemented to Methocult H4035. As positive control, CD34+ stem cells were seeded in MethoCult H84434 (includes EPO, Stem Cell Technologies). Colony-forming units (BFU-E; colonies >50 cells, CFU-E; colonies <50 cells, CFU-G/M/GM and CFU-GEMM) were automatically analysed and counted after 14 days of incubation at 37 °C using STEMvision equipment (Stem Cell Technologies). CFUs were examined in duplicates.

### Hypoxia in primary hippocampal neurons

Hippocampal neurons were prepared from new-born Wistar-Imamichi rats according to Lewczuk^44^ with slight modification. Briefly, hippocampi were dissected in HBSS solution supplemented with 0.1% D-Glucose, 1 mM sodium bicarbonate, 1 mM pyruvate and 10 mM HEPES, and transferred into precooled HBSS/B27 solution (0.2% B27) until trituration. Hippocampi were incubated for 30 min with papain (4 ml, 184 U/ml) at 30 °C and shaken every 5 min. 25 µl of DNase I (10 mg/ml in HBSS) were added for 2 min. The hippocampi were triturated in HBSS/B27 and the cell suspension was underlayered with a density gradient prepared in four 1 ml steps of 35, 25, 20 and 15% of Optiprep (Thermo Fisher Scientific) in HBSS/B27. The cell suspension was centrifuged for 15 min (2000 rpm, RT). Neuron-enriched fractions were collected and washed with 5 ml HBSS/B27. After centrifugation at 1100 rpm for 2 min the pellet was resuspended in growth medium (1 ml; NeurobasalA, 0.2% B27, 5 ng/ml bFGF, 0.5 mM L-glutamine, 100 U/ml penicillin and 100 µg/ml streptomycin). The neurons were plated on poly-D-lysine coated 12 mm coverslips in 4-well-plates, with a density of 8000 cells/coverslip. At day 3 the medium was replaced by starvation medium (growth medium without bFGF and B27) complemented with EPO 3 U/ml (NeoRecormon, Roche, UK), EV-3 at 0.3 U/ml and 3 U/ml weight equivalent of EPO, or placebo (solvent solution). At day 5, cultures were exposed to hypoxia or normoxia for 20 hours. Cultures were stained with 0.4% Trypan blue. Total cell number and number of dead cells were counted. Results are presented as % cell death; the hypoxia effect was set to 100%.

### Animals

Juvenile (28 day-old) male *C57BL/6NCrl* mice were used. They were housed in groups of 5 in standard plastic cages and maintained in a temperature-controlled environment (21–22  °C) on a 12 hours light/dark cycle with food and water available ad libitum. All experiments were approved by and conducted in accordance with the regulations of the local Animal Care and Use Committee (Niedersächsisches Landesamt für Verbraucherschutz und Lebensmittelsicherheit—LAVES).

### Treatment

Mice (10–11 per group) were injected intraperitoneally (i.p.) with recombinant human erythropoietin (EPO) (NeoRecormon, Roche, 5000 IU/kg body weight), EV-3 (IBA, weight equivalent) or placebo (solvent solution), each at a volume of 0.01 ml/g. Treatment started at the age of 28 days and continued every other day for 3 weeks (11 injections in total). After the last injection, the animals were adapted for 1 week to the food deprivation procedure (85% of free feeding body weight) prior to start of the touch-screen experiment.

### Haematocrit determination

A separate cohort of mice either received EPO (n = 15), EV-3 (n = 14) or placebo (n = 15) injections as described above. Mice were tested for haematocrit levels 9 days after the last injection. Blood samples were collected in EDTA Microvettes, transferred into heparinized capillaries, centrifuged and the volume percentage of erythrocytes in whole blood was then determined using a haematocrit reading device. T-tests for independent samples were used to compare haematocrit levels between groups.

### Cognitive testing (Touch-screen visual discrimination task)

Detailed description of the operant chambers and experimental procedures including visual stimuli have been described previously^45^. In brief, every mouse was subjected to the following sequence of test phases: (1) acclimation, (2) autoshaping, (3) pre-training 1–3, and (4) visual discrimination. The daily sessions were terminated after 30 min or if the mouse had accomplished the specific learning criterion of the respective test phase. Acclimation: The animal learned that food pellets are available in the food magazine. Learning criterion: Collection of 10 pellets within 30 min. Autoshaping: The animal learned to associate the presentation of visual stimuli on the touch-screen with the delivery of food pellets. Learning criterion: Collection of 30 pellets within 30 min. Pre-training 1: The animal learned that a touch response to the correct side of the screen, i.e. where a visual stimulus was presented, is rewarded by the delivery of a food pellet. Learning criterion: 30 correct responses within 30 min. Pre-training 2: Now, the animal had to learn to initiate a new trial by disrupting the infrared light beam in the magazine a second time (after it had collected the food pellet). Pre-training 3: During this stage the animal had to memorize that an incorrect response resulted in a 5 sec timeout and the initiation of a correction trial. Learning criterion: At least 27 correct responses out of 30 trials. Visual discrimination: Here, the animal had to discriminate between a rewarded visual stimulus S+ (correct response rewarded with food pellet) and a non-rewarded stimulus S- (incorrect response resulted in timeout). Learning criterion: At least 27 correct responses out of 30 trials or 26 correct responses out of 30 trials on 2 consecutive days, whatever appeared first. The following performance readouts were measured: 1. Days needed to achieve the pre-training criteria (1–3), 2. Days needed to achieve the learning criterion in the visual discrimination task, 3. Days needed to complete whole sequence of tasks (pre-training and visual discrimination). **Statistics:** Touch-screen data were analysed by means of Kaplan-Meier analysis. Two-tailed p-values lower than 0.05 were considered to be significant.

## Results

### Alternative splicing of *EPO* in human tissue confirmed

**Figure 1:**
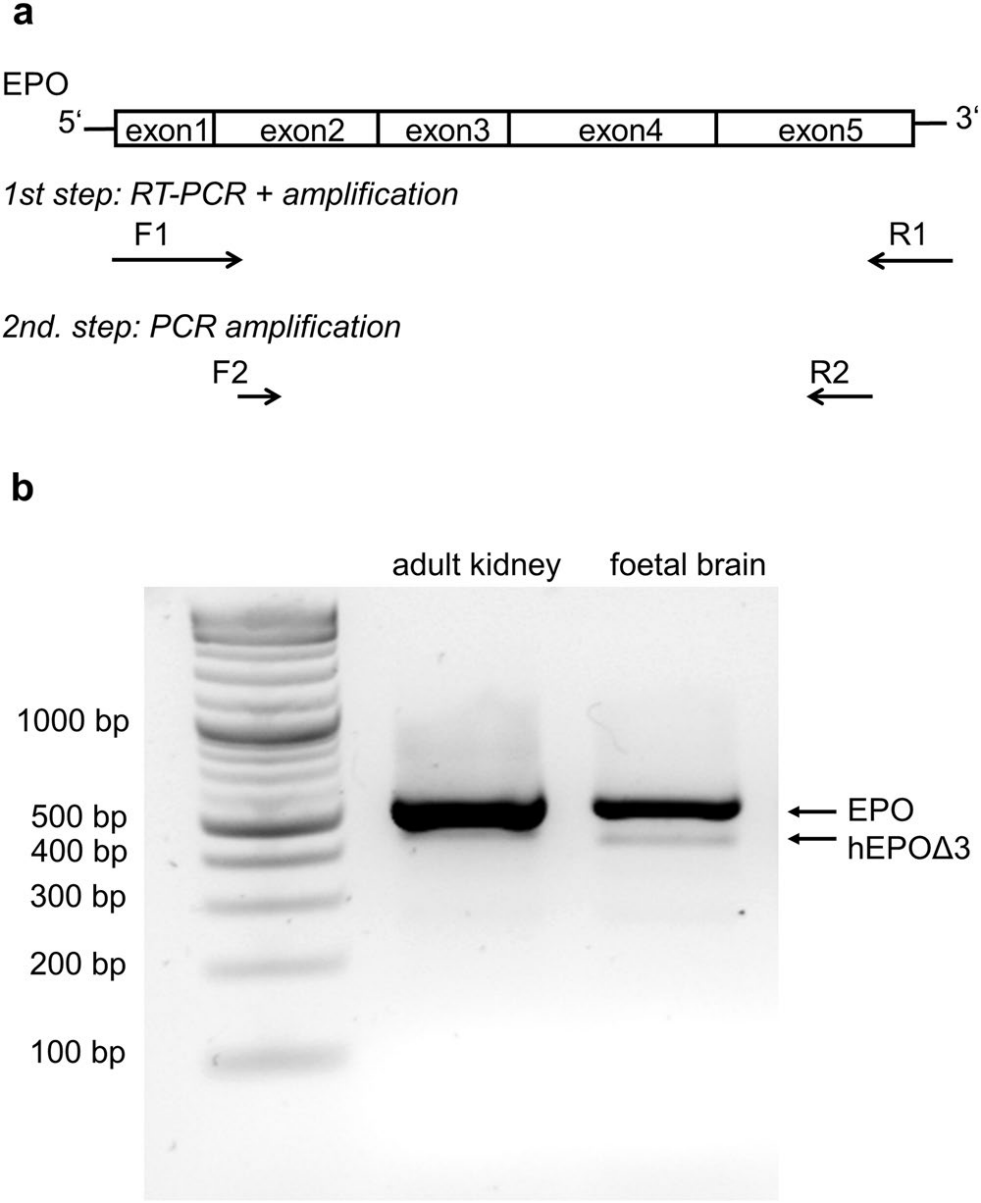
Isolation of the human EPO spliced transcript hEPOΔ3. (**a**) Reverse transcription and PCR amplification is performed with a set of primers for full-length amplification of *EPO* lying 5′ and 3′ of the coding sequence in exon 1 and exon 5. Then some product is taken for re-amplification with an internally-situated set of primers lying in exon 2 and exon 5. (**b**) Agarose gel of *EPO*-PCR amplification products from human adult kidney and foetal brain reveals two products at 550 bp and 460 bp, respectively.

At baseline condition, *EPO* mRNA is expressed at very low levels^46, 47^. Therefore, we used a two-step PCR approach to amplify *EPO* mRNA from human foetal brain and human kidney total RNAs. Primer sequences for reverse transcription and first PCR amplification were taken from the original discovery description^19^ lying at 5′ (exon 1) and 3′ (exon 5) borders of the coding region. A second primer pair lying in exon 2 and exon 5 was used in a second PCR, using products from the first PCR as template (Fig. 1a). Electrophoretic separation of 2-step PCR amplification products revealed the expected PCR product at approximately 550 bp corresponding to *EPO* truncated by 17 nucleotides at 5′ and by 16 nucleotides at 3′ through the usage of the second primer pair lying in exon 2 and exon 5 of the EPO gene. A second PCR product at approximately 460 bp corresponding to the 495 bp product described before^19^ was observed in both adult kidney and foetal brain samples (Fig. 1b). The amplification products were cut from the agarose gel and purified separately. Subcloning and sequencing confirmed the previously reported results^19^, i.e. revealed the *EPO* full-length form for the long PCR products and a shorter *EPO* form containing an internal 87-nucleotide deletion ranging from nucleotide 160 to 246. The human *EPO* gene consists of five exons and four introns. The missing nucleotides in the short *EPO* transcript (hEPOΔ3) correspond to exon 3. The hEPOΔ3 transcript is seemingly generated through alternative splicing using the AG dinucleotide at the 3′ end of exon 3 as alternative splice acceptor site^17^.

### Expression of the hEPOΔ3 transcript is upregulated in disease conditions

**Figure 2:**
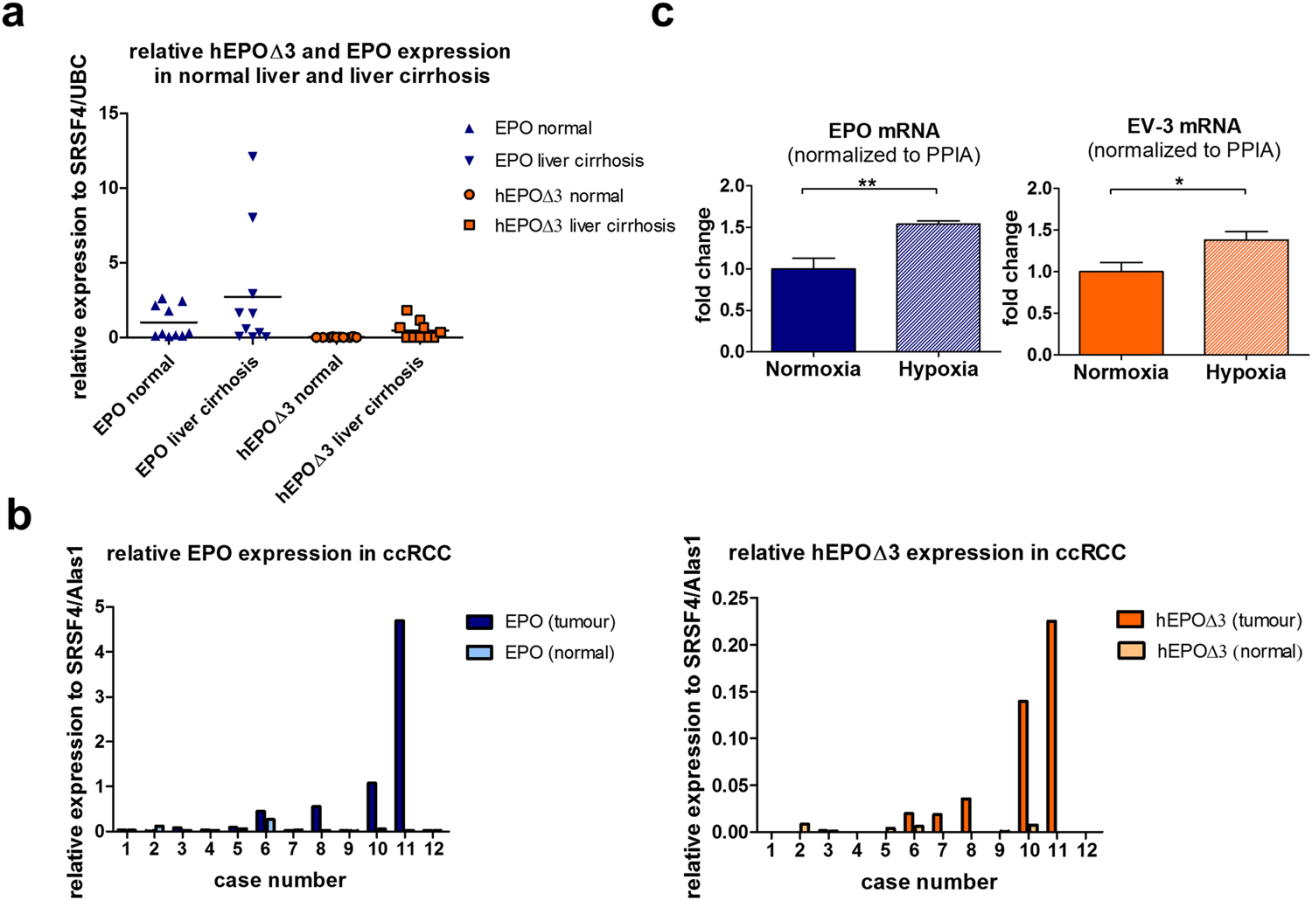
Expression analysis of EPO and hEPOΔ3 transcripts in human tissues and the human cell line HepG2 using quantitative PCR. (**a**) Normal and cirrhotic liver FFPE samples (n = 10 per condition) were analysed, using UBC and SRSF4 as reference genes. *EPO* mRNA expression is detectable in both normal (▲) and cirrhotic liver tissue (▼), individual cirrhotic samples show 3–6 fold increase compared to mean of normal tissue. hEPOΔ3 is only amplified from cirrhotic livers (□) but not from normal livers (○). (**b**) *EPO* and hEPOΔ3 expression was analysed in clear cell renal cell carcinoma FFPE samples and compared to adjacent normal tissue (n = 12 per condition). Expression levels of SRSF4 and Alas1 were constant in tumour and matched normal tissue and served as reference genes. *EPO* expression is enhanced in clear cell renal cell carcinoma samples 8, 10 and 11 compared to adjacent normal tissue. These samples show also highest hEPOΔ3 expression. (**c**) *EPO* and hEPOΔ3 expression is induced in HepG2 cells exposed to hypoxic conditions (1% O_2_) for 4 h. Cyclophilin A expression was not affected by hypoxia and used as internal control gene. (n = 4); two-tailed t-test; mean ± SEM presented.

Alternative splicing is often modulated according to cell type or in response to external stimuli^48^. Therefore, in a pilot study we studied tissue expression of *EPO* and hEPOΔ3 transcripts in a heterogeneous cDNA panel derived from commercially available RNA samples using quantitative PCR. Within this panel, high *EPO* and hEPOΔ3 expression was found in one liver cirrhosis sample (see Supplementary Fig. S1). In order to substantiate this observation, we broadened our study to a panel of 10 healthy liver samples and 10 cirrhotic liver samples, all formalin fixed paraffin embedded (FFPE), using UBC and SRSF4 as reference genes^49, 50^. We designed SYBR-green-based quantitative PCR assays specific for the *EPO* full-length form or for hEPOΔ3. In normal liver tissue, *EPO* expression was detectable whereas hEPOΔ3 was below detection limit. In cirrhotic liver tissue, the analysis showed a considerably high inter-individual variation in the expression levels of both *EPO* and hEPOΔ3. In individual samples, the mean expression level of *EPO* was 2.7-fold higher in liver cirrhosis samples compared to normal liver tissue (Fig. 2a). Expression of the hEPOΔ3 form was detectable in those cirrhotic liver samples which also showed enhanced *EPO* expression. Suggesting therefore co-regulation of *EPO* and the spliced transcript hEPOΔ3 in diseased condition, we further studied expression profiles in a panel of clear cell renal cell carcinoma (ccRCC) samples and adjacent normal renal tissue. In this panel, *EPO* expression was enhanced in 3/12 tumour samples compared to adjacent normal tissue. Again, these samples showed also highest hEPOΔ3 expression (Fig. 2b). EPO expression is regulated by a distinctive oxygen-sensing mechanism involving the HIF pathway^51^. The human EPO-producing liver hepatocellular carcinoma cell line HepG2^37^ is an established model to study hypoxia-inducible EPO transcription (e.g. 52, 53). Thus, we assessed the change in *EPO* and putative hEPOΔ3 transcripts in HepG2 cells exposed to hypoxia (1% O_2_) for 4 hours. UBC, SRSF4 and cyclophilin A (PPIA) were tested for hypoxic response. As PPIA expression was stable under hypoxic conditions, being in line with observations from others^52, 54^, it was used as internal control. Importantly, we did not only detect hEPOΔ3 in HepG2 cells, but *EPO* and hEPOΔ3 were both hypoxia inducible in HepG2 cells (Fig. 2c). In summary, our data show concomitant up-regulation of *EPO* and hEPOΔ3 under hypoxia and in diseased condition.

### Protein structure prediction/modelling

**Figure 3:**
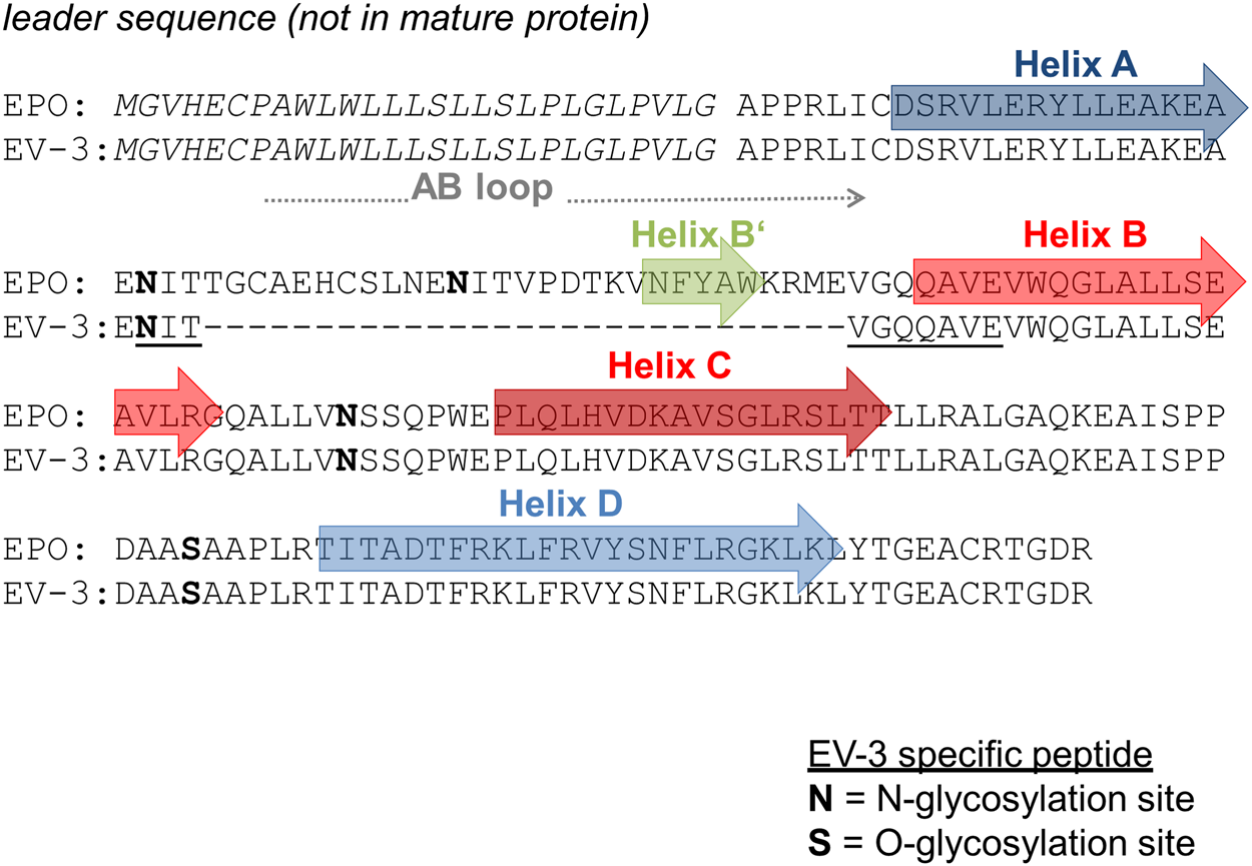
Depiction of the EV-3 specific peptide used for nano-LC-ESI-MS/MS analysis. According to the obtained sequence of the two *EPO*-PCR amplification products from human adult kidney and foetal brain, aminoacid sequences of EPO and hEPOΔ3 derived protein EV-3 differ only by 29 aminoacids. The deleted region contains one N-glycosylation site and the AB loop including the minihelix B’. The underlined amino acids in the EV-3 sequence represent the EV-3 specific peptide used in nano-LC-ESI-MS/MS. The representation of EPO’s primary structure is taken from Boissel *et al*.^84^.

Human EPO is a heavily glycosylated protein consisting of one 165 amino acid chain and containing three N-linked (Asn-24, Asn-38, Asn-83) and one O-linked (Ser-126) oligosaccharide side chains^55, 56^ (Fig. 3). The carbohydrates contribute about 40% of the total molecular weight^57^. EPO and the hEPOΔ3 derived EV-3 protein differentiate by 29 amino acids^19^ (Fig. 3) leading to a reduction in molecular mass of unglycosylated proteins from 20 kDa for EPO to 16.8 kDa for EV-3. In the glycosylated form of the two proteins, the loss of one N-glycosylation site (Asn-38) in EV-3 leads to a more distinct difference in apparent molecular weight (~5–8 kDa). The human EPO protein has a four-helix bundle structure^58^, which is typical for haematopoietic growth factors. The four long α-helices (A, B, C and D) are connected by two long cross-over loops (AB and CD) and one short loop (BC) (see Supplementary Fig. S2). Near the carboxy end of the AB loop is a short alpha-helical segment (minihelix B’) which is important for binding to the EPO-receptor^58^. As previously described, the EV-3 protein sequence suggests loss of the AB loop^19^. Using automated homology modelling this is predicted to result in a considerable change in tertiary structure compared to EPO (see Supplementary Fig. S2).

### The hEPOΔ3 derived protein EV-3 is isolated from HepG2 culture supernatants using immunoprecipitation

**Figure 4:**
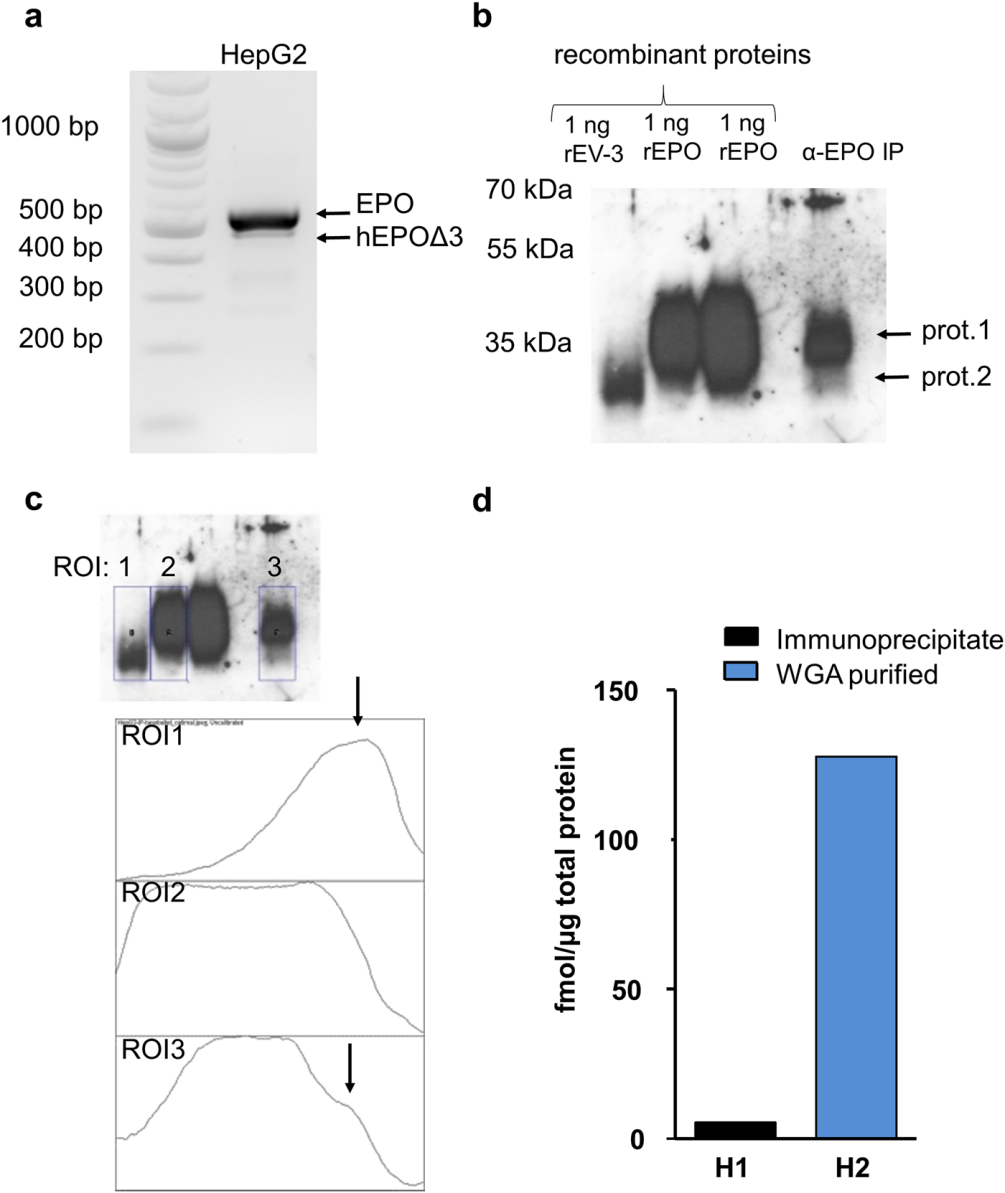
Isolation of hEPOΔ3 from HepG2 cells and detection of hEPOΔ3 derived protein EV-3 and EPO in HepG2 cell culture supernatant. (**a**) The nested PCR approach amplifies EPO full-length and hEPOΔ3 transcripts from HepG2 cells. (**b**) Captured proteins of HepG2 supernatants (α-EPO IP, using biotinylated anti-EPO antibody BAF959) are visualized by western blotting using a polyclonal rhEPO-antibody (H-162). The Western Blot shows two EPO-antibody reactive proteins (prot. 1 and prot. 2) running at the same size of recombinantly produced EV-3 (rEV-3) and EPO (two different batches of rEPO). (**c**) Densitometry analysis was done on the Western Blot image (jpeg) shown in Fig. 4b. The plot profile of the HepG2 immunoprecipitate in lane 5 (ROI3) reveals a faint but distinct second peak, corresponding to the plot profile of EV-3 loaded in lane 1 (ROI1). (**d**) EV-3, indicated by the specific peptide ‘DITVGQQAVE’, was detected in HepG2 supernatants by nano-LC-ESI-MS/MS. Quantification of EV-3 specific peptide (fmol/µg total protein of analysed sample) in sample H1, the immunoprecipitate (α-EPO-IP) of HepG2 supernatant, or sample H2, HepG2 supernatant enriched for glycoproteins by WGA chromatography.

We intended to provide evidence for the expression of the hEPOΔ3 derived EV-3 protein. Therefore, we used the HepG2 cell line which expresses *EPO* and hEPOΔ3 transcripts (Fig. 4a). If we find that hEPOΔ3 transcript is translated into protein, we assumed that it is secreted by the HepG2 cells similarly to EPO. Thus, supernatants of HepG2 cultures were pooled and concentrated and a biotinylated polyclonal antibody specific to the N-terminus of EPO was used for immunoprecipitation. Recombinant produced proteins, rEV-3 and rEPO, were used as positive controls for Western Blot. EPO and EV-3 migrate as diffuse bands with apparent molecular masses of approximately 40 kDa (EPO) and 35 kDa (EV-3). In the supernatants of HepG2 cultures, the polyclonal anti-EPO antibody precipitated two proteins running at the size of EPO and EV-3 (Fig. 4b,c). The identity of the two proteins was confirmed through mass spectrometry as described below.

### Confirmation of EV-3 protein in HepG2 supernatant using multiple-reaction monitoring

Systematic processing of EPO and EV-3 containing samples, as described in the method section, allowed for the generation of two peptides enabling the identification and differentiation of EPO and EV-3 within one sample. The resulting peptide with the sequence ‘DITVGQQAVE’ is contained in EV-3 but not in EPO (Fig. 3) and no other protein was found to include that sequence; therefore this peptide is the specific identifier of EV-3. Peptide sequence ‘DITTGCAE’ is absent in EV-3 and contained in human EPO only and therefore serves as the specific identifier of EPO in the processed samples (Table 1).

The protein samples obtained from HepG2 supernatants by either immunoprecipitation with an anti-EPO antibody or WGA purification were processed (PNGase F and Asp-N/Glu-C digestion). Thereby, EV-3 or EPO proteins in the sample are cleaved leading to the generation of above mentioned identifier peptides of EV-3 and EPO. For identification and quantification through multiple-reaction-monitoring (MRM), isotopically labelled EV-3 and EPO identifier peptides were spiked into the analyte (processed sample) and endogenous peptides were identified through their identical retention time. The EV-3 specific peptide DITVGQQAVE was reliably detected in the samples; also peptide DITTGCAE (specific for EPO) was detected. This finding confirms the presence of EV-3 and EPO in the supernatants of HepG2 cells as indicated by the Western Blot (Fig. 4b,c).

Quantitation of EV-3 by its specific peptide was robust, whereas quantitation of EPO by its specific peptide was unstable: DITTGCAE was detected but yielded standard curves only for 1/5 transitions and therefore, parallel quantitation of EPO in the same sample was disabled. The EV-3 specific peptide DITVGQQAVE was quantified with 5/5 transitions and revealed that the level in the immunoprecipitate (H1; 5.5 fmol/µg of total protein) was considerably lower than in the WGA purified HepG2 supernatant (H2; 127.7 fmol/µg of total protein) (Fig. 4d). Quantification is relative to the amount of total protein in the processed sample. Immunoprecipitation and lectin-affinity chromatography are two different methods to enrich for EPO and EV-3, but neither approach will result in a pure EPO/EV-3 sample. Whereas the elution step in the immunoprecipitation approach co-elutes the anti-EPO antibody, WGA purification also enriches other glycoproteins, thereby explaining the different relative levels of EV-3 specific peptide quantified in the samples. In summary, the analysis of HepG2 supernatants by MRM shows that hEPOΔ3 expressing HepG2 cells produce and secrete the corresponding EV-3 protein.

### EV-3 protein is detected in human serum

**Figure 5:**
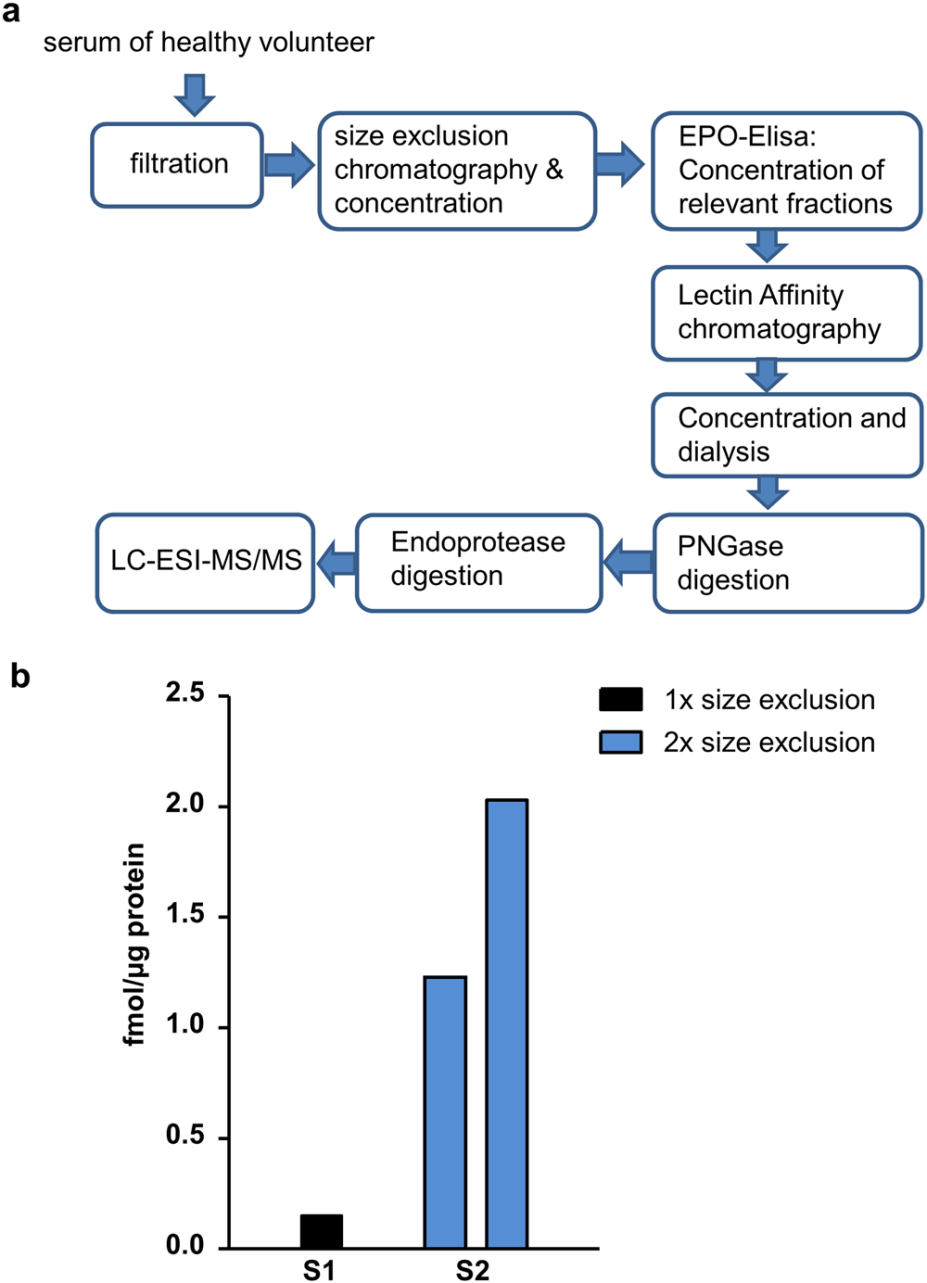
EV-3 protein identification in human serum from healthy donors. (**a**) Depiction of the optimized multi-step purification strategy of serum for the detection and quantification of the EV-3 specific peptide by nano-LC-ESI-MS/MS. (**b**) In serum samples of the healthy human donors (S1, S2), the EV-3 specific peptide ‘DITVGQQAVE’ was detected by using nano-LC-ESI-MS/MS. Applying two size exclusion chromatography steps to serum S2 enriched EV-3 in the sample. Compared to S1, higher levels of the EV-3 specific peptide (fmol/µg total protein) were quantified in two fractions of S2.

The above described determination of the *EPO* splice variant hEPOΔ3 as well as EV-3 protein in HepG2 cells depicts a critical result of our study; however, cell lines have some limitations regarding the representation of an *in vivo* situation. Therefore, we investigated blood from healthy human donors for the presence of EV-3 protein. A multi-step purification strategy (Fig. 5a) including size-exclusion chromatography and glycoprotein affinity chromatography was developed for processing human serum in order to enrich for EV-3 and EPO in the fractions used for analysis (see Supplementary Figs S3 and S4). Mass spectrometry analysis of the purified and processed samples was performed according to the previously established MRM protocol. Serum samples of two healthy donors were examined and in both of them the EV-3 specific peptide ‘DITVGQQAVE’ was identified, indicating the presence of EV-3 protein (Fig. 5b). The level of EV-3 in sample 1 (donor 1), with one size exclusion chromatography included in the processing of the sample, was 0.15 fmol/µg total protein. As the applied technology allows loading of only 3.5 µg total protein/test sample, we sought to further reduce the amount of larger proteins like albumin (66.5 kDa) by additional size exclusion chromatography and thereby enrich for EV-3 and EPO in the test sample. Thus, processing of sample 2 (donor 2) included two size exclusion steps. Two different fractions of the fully processed serum sample were analysed and the levels for the EV-3 specific peptide were 1.24 fmol/µg and 2.03 fmol/µg in the respective test sample. The EPO-specific peptide DITTGCAE was also detected in the test samples but only in 1/5 transitions and therefore was unqualified for quantification.

The here described detection of EV-3 in human serum is the first proof that human hEPOΔ3 is translated into protein and secreted into the blood circulation.

### EV-3 has no erythropoietic colony forming unit (CFU-E) properties in human CD34+ progenitors

**Figure 6:**
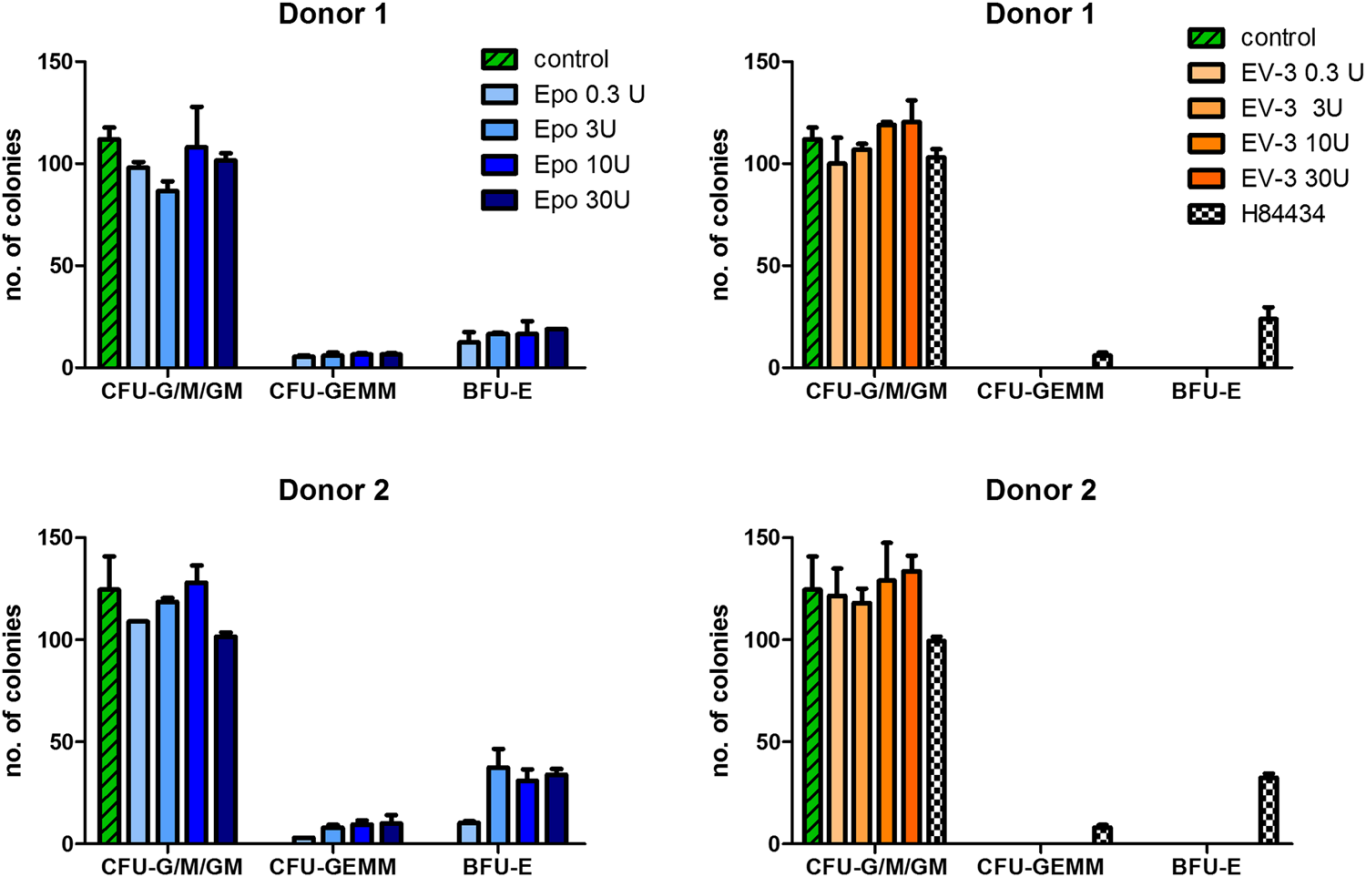
EPO but not EV-3 promotes formation of erythroid progenitors from human CD34+ haematopoietic stem cells. CD34+ cells (500 cells/well) from two donors were cultured in EPO-free MethoCult medium supplemented either with EPO or EV-3 at different concentrations (0–30 U/ml) for 14 days. Analysis of the different colony-forming units (CFU) revealed no erythroid progenitors (CFU-GEMM or BFU-E) in control or EV-3 treated wells but in wells treated with EPO or positive control H84434 (medium includes EPO). n = 2 per treatment; CFU-G/M/GM, colony-forming unit-granulocyte or -macrophage or mix of granulocyte and macrophage; CFU-GEMM, colony-forming unit granulocyte, erythrocyte, monocyte, megakaryocyte; BFU-E, burst-forming unit erythroid.

CD34 is a marker of haematopoietic stem- and progenitor cells^59, 60^. The proliferation and differentiation of HSC depends on a variety of cytokines, the most prominent for the erythroid pathway being erythropoietin. In a semisolid colony forming unit assay, early progenitor cells will yield larger colonies referred to as burst forming units, while progenitors further down the differentiation path will yield smaller colonies (CFU-E). While complete Methocult medium (H84434, comprising EPO) yielded BFU-E and CFU-E at numbers representative for the CD34 source and content of the specimen (Fig. 6), medium that is devoid of EPO (H4035) as expected failed to show any formation of erythroid colonies. A systematic analysis of the EPO dose necessary to yield a maximum of erythroid colonies showed a clear correlation of the EPO concentration with the number and phenotype of erythroid colonies with a maximum erythroid colony stimulating capacity at 3 U/ml. Interestingly, EV-3 was not able to replace EPO in the CFU Assay. Even at concentrations 10 times higher than the optimum concentration of EPO, EV-3 did not yield any erythroid colonies (Fig. 6). Since CD34+ haematopoietic stem cells as well as BFU-E and CFU-E express the EPOR^61, 62^, the absence of erythroid colony forming stimulating effect of EV-3 on erythroid progenitors might therefore be due to the absence of the high affinity binding site to the EPOR in the EV-3 splice variant of erythropoietin.

### Neuroprotective effects of EV-3 against hypoxia-induced cell death confirmed

**Figure 7:**
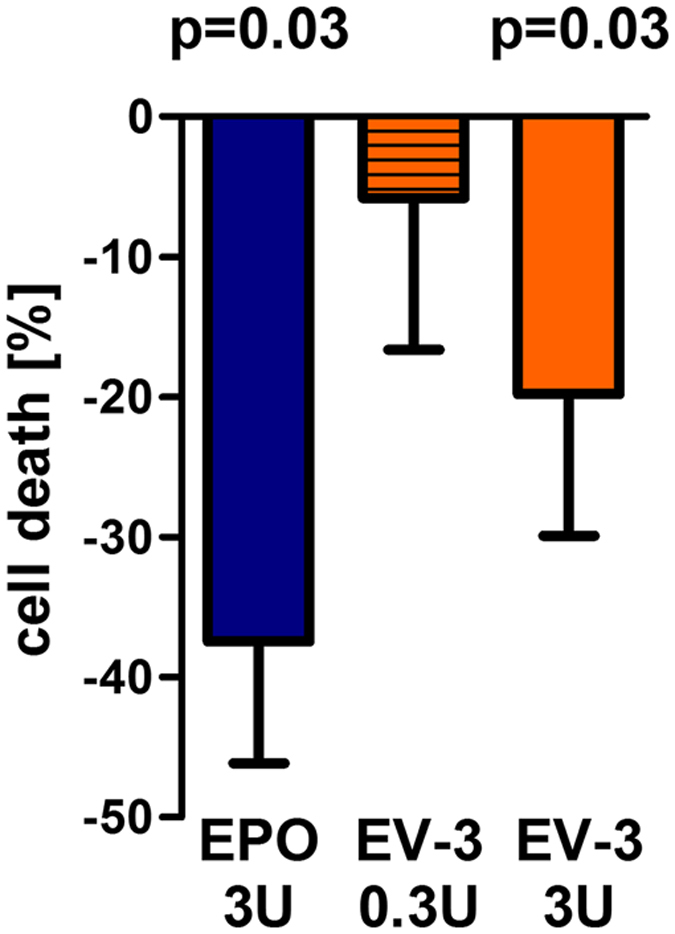
EV-3 and EPO protect primary hippocampal neurons from cell death. Pretreatment with EPO or EV-3 reduced cell death, induced by starvation (48 h) followed by hypoxia (20 h), of rat primary hippocampal neurons (n = 5/treatment; hypoxia effect set to 100%; one-tailed paired t-test, EPO and EV-3 [U weight equivalents] treatment compared to placebo). Mean ± SEM presented.

EPO’s protective effects against hypoxia-induced cell death is well described in the literature^44, 63, 85^. We sought to investigate previously reported conservation of neuroprotective properties in the naturally occurring EPO-isoform EV-3^19^. Rat primary hippocampal neurons were cultured and hypoxia-induced cell death, indicated by the percentage of Trypan blue- positive cells of all cells in the respective well, under placebo conditions (control) were set as 100%. Pre-treatment with EPO (3 U/ml) lead to reduction of hypoxia-induced cell death (p = 0.03, compared to control). Pre-treatment with EV-3 (weight equivalent to 3 U/ml of EPO) also significantly protected rat primary hippocampal neurons against hypoxia-induced cell death (p = 0.03, compared to control), although the total effect seems slightly lower than that of EPO (Fig. 7). The results point towards the preservation of neuroprotective effects in EV-3.

### EV-3 facilitates cognitive performance of mice and is devoid of haematocrit increasing properties

**Figure 8:**
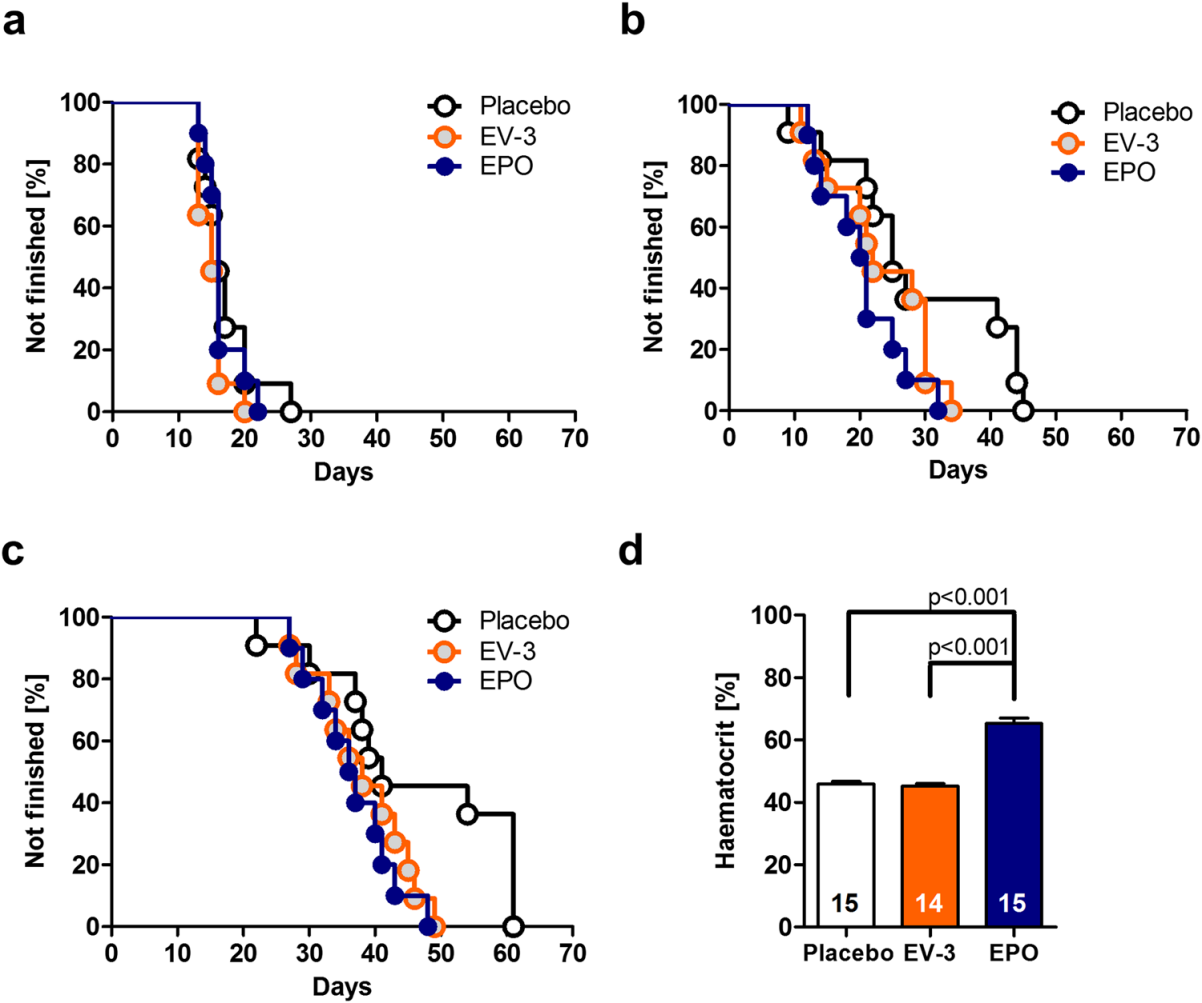
Cognitive performance in a touchscreen-test and haematocrit levels in placebo, EV-3 and EPO treated mice. Kaplan-Meier presentation of group performance during (**a**) pretraining 1–3, (**b**) visual discrimination and (**c**) overall task (pretraining and visual discrimination). Curves represent group performance (n  =  10–11 per group) and indicate the cumulative percentage of animals not reaching performance criteria of the touch-screen paradigm. The EPO- and the EV-3 treated mice finished the overall task (**c**) 10 days earlier than the placebo group (EV-3 vs. placebo, p = 0.060, EPO vs placebo, p = 0.041*). (**d**) Following 3-weeks treatment with EPO (5000 IU/kg, NeoRecormon) versus EV-3 (weight-equivalent dose) and placebo, haematocrit is significantly enhanced in the EPO-treated but not in the EV-3 treated group.

Putative *in vivo* actions of EV-3 were tested in mice. A learning task was chosen as enhancement of cognitive function in mice was reported for EPO as well as for its carbamoylated derivative^64, 65^. Cognitive performance in a visual discrimination task^45, 66^ of EPO, EV-3 and placebo-injected mice was tested in a touch-screen based operant system. Pretraining performance was not significantly different between the placebo, EV-3 and EPO groups (Kaplan-Meier analysis: Placebo vs. EV-3: χ^2^ = 2.057; p = 0.152, Placebo vs. EPO: χ^2^ = 1.83; p = 0.669, EV-3 vs. EPO: χ^2^ = 1.674; p = 0.196; Fig. 8a). During the visual discrimination task, EPO-treated mice showed significantly faster learning as compared to the placebo-treated mice (χ^2^ = 4.178; p = 0.041; Fig. 8b). No significant difference was found between EPO and EV-3 treated mice (χ^2^ = 1.344; p = 0.246). Furthermore, we investigated whether the overall performance in which pretraining and visual discrimination phases were combined was different between groups. EPO-treated mice showed a significantly better overall performance as compared to the placebo-treated mice (χ^2^ = 4.177; p = 0.041; Fig. 8c). Interestingly, EV-3 treated mice performed similarly to EPO treated mice, resulting in a close-to-significant difference between EV-3 and placebo-treated mice (χ^2^ = 3.541; p = 0.060). EV-3 mice were faster in the acquisition of the different stages of the touchscreen task compared to placebo. No such difference was found for the pair-wise comparison of the EPO and EV-3 groups (χ^2^ = 0.503; p = 0.478). These results suggest that touchscreen performance is facilitated by both EPO and EV-3 treatment.

In a second batch of mice, injected according to the treatment scheme of the animals used for the cognitive testing, we investigated the erythropoietic properties of EV-3 by determination of the haematocrit. No difference between haematocrit levels of EV-3 and placebo-treated mice (Fig. 8d) was found (T[27] = 0.67; p = 0.509). Haematocrit levels were increased in EPO-treated mice as compared to both EV-3 (T[27] = 10.27; p < 0.0001; t-test for independent samples) and placebo treated mice (T[28] = 10.29; p < 0.0001). These results show that pro-cognitive effects of EV-3 on touchscreen performance are not related to changes in haematocrit levels. Further, EV-3 differs to EPO in terms of erythropoietic properties.

## Discussion

The present study evaluates the existence and functionality of a naturally occurring variant of EPO. The finding of the *EPO* splice variant lacking exon 3 (hEPOΔ3), originally described by Meisel, Priller, Bonnas and Dirnagl (Charité Berlin)^19^, in human foetal brain and kidney were confirmed. We further report the co-regulation of *EPO* and hEPOΔ3 in the human cell line HepG2 as well as in human tissue and importantly, the detection of the hEPOΔ3 corresponding protein-product EV-3 in human serum. Investigating the biological functionality of EV-3, we provide evidence that recombinant EV-3 does not promote erythroid progenitors but shares EPO’s neuroprotective properties. Our first *in vivo* studies with EV-3 indicate beneficial effects on cognitive performance and simultaneously show that EV-3 is a non-erythropoietic variant of EPO expressed in humans.

The discovery of alternatively spliced variants of human *EPO* together with the indicated functionality of the protein product of hEPOΔ3, i.e. loss of erythropoietic but preservation of neuroprotective properties, has only recently been described. One aim of our studies was to broaden the spectrum of human samples used for expression analysis of the splice variant hEPOΔ3. Our results revealed that expression of hEPOΔ3 is in accordance with *EPO* expression^2–6^: The hEPOΔ3 transcript was detected in samples of kidney, liver and foetal brain but seems to be expressed at much lower levels than *EPO*. In a subset of human cirrhotic liver tissue samples, we see enhanced expression of both, *EPO* and hEPOΔ3. Although elevated blood EPO concentrations are not generally found in patients with cirrhosis^32, 67^, local hepatic tissue hypoxia occurs in patients with cirrhosis^68^. Thus, enhanced *EPO* and hEPOΔ3 expression in liver cirrhosis is likely explained by hypoxic mechanisms and the observed parallel enhancement in individual samples implies that *EPO* and hEPOΔ3 transcript expression are co-regulated *in vivo*. This is further supported by our finding of concomitantly enhanced *EPO* and hEPOΔ3 transcript expression in ccRCC samples. Several studies reported an increased expression of *EPO* as well as *EPOR* in human ccRCC, the question of whether the EPO/EPOR pathway plays a functional role and correlates to prognosis in ccRCC is under investigation by others^35, 69^. In line with the findings in human tissue, we detected enhanced expression of *EPO* and hEPOΔ3 *in vitro* in HepG2 cells exposed to hypoxic conditions. Oxygen-dependent regulation of the EPO gene is organ-specific (reviewed in 39, 70). In the liver, hypoxia inducible elements are located within 0.4 kb 5′ and 0.7 kb 3′ of the EPO gene and sequences required for inducible expression in the kidney are located greater than 6 kb 5′ or 0.7 kb 3′ to the gene^71^. Thus, from the gene structure, hypoxia inducible elements and the promoter region are not supposed to be affected by alternative splicing of exon3 in hEPOΔ3. The observed co-regulation of the *EPO* and the hEPOΔ3 transcript in HepG2 cells and in human tissues implicates usage of identical promoter and hypoxia inducible elements. So far, our observations together with reports regarding cytoprotective action of EPO on acute ischemia-induced injury of liver or kidney^72–76^ lead us to hypothesize that the enhanced *EPO* and hEPOΔ3 transcript expression is part of a response playing a crucial role for endogenous tissue protection.

The detection of a splice variant co-regulated with the origin hardly allows concluding the translation into an active protein. Therefore, we aimed at the detection of hEPOΔ3 corresponding protein EV-3 protein in human samples. Additionally, we used the HepG2 cell line, where we have detected the hEPOΔ3 transcript, for further studies. A multistep processing protocol was established which allowed the clear separation of EPO and EV-3 and the detection by means of multiple reaction monitoring. In the supernatants of HepG2 cells we detected the peptide exclusive for the protein-product EV-3 derived from the hEPOΔ3 transcript. The respective peptide sequence covers the amino acids encoded by the end of exon 2 and by the beginning of exon 4 of the *EPO* gene and is specific for the product of the splice variant hEPOΔ3, lacking exon 3. More importantly, we also detected EV-3 in serum of healthy humans. Here, additional purification steps needed to be included before analysing the samples by MRM because of the high abundancy of proteins in human serum. Nevertheless, we could identify the EV-3 specific peptide in both serum samples investigated. This finding allows the conclusion that the hEPOΔ3 transcript, detected in human specimen, is translated into EV-3 protein and is secreted into the blood circulation in healthy individuals. This is a strong indicator for an endogenous function of this protein identified in humans.

Similarities in the expression and regulation of *EPO* and hEPOΔ3 lead us to the assumption that biological functions of EV-3 and EPO might overlap to some extent. Starting to investigate the biological function of EV-3, we refer here to well documented effects of EPO and investigated the EV-3 effects upon (i) erythropoiesis, (ii) hypoxia-induced neuronal cell death^7^, and (iii) cognitive performance in a mouse long-term learning paradigm^66^. Our results in cultured human CD34+ showed that EV-3, contrary to EPO, does not promote erythroid progenitors (BFU-E or CFU-E) in the early phase of erythropoiesis. CD34+ haematopoietic stem cells as well as BFU-E and CFU-E have been reported to express the EPOR^61, 62^. We also observed that repeated administration of EV-3 to mice is without influence on haematocrit levels. Together, this indicates that EV-3 does not have the binding properties like EPO in order to interact with the EPOR homodimer during the crucial steps of erythrocyte differentiation. This is in line with the EV-3 protein sequence^19^ which suggests loss of the ‘AB’ cross-over loop present in the EPO protein (Fig. 3). The ‘AB’ loop contains a segment which has been identified as being important for binding to the EPOR and the formation of the homodimer complex^58, 77^. Currently, we have no information on the receptor(s) binding EV-3. On the other hand, we observed a dose-dependent protective effect of EV-3 on primary hippocampal neurons *in vitro*. Compared to the effects of EPO in this assay system, the efficacy of EV-3 at a similar dose as EPO seemed to be slightly lower. In terms of biological function of EV-3, we also show here that recombinant EV-3 is active *in vivo* and improves, again with a slightly lower effect than EPO, cognitive performance of mice in a long-term learning paradigm. The slight difference we observed in a preliminary pharmacokinetic study (see Supplementary Fig. S5) unlikely explains the somewhat weaker effects of EV-3. The fact that the statistical significance in the cognition test was just missed (p = 0.06) may be due to a still suboptimal dosing regimen of EV-3 which might be different to EPO and needs to be further investigated. Clearly, information regarding the receptor and signalling pathways will help to optimize the protocols for testing the effects of EV-3.

The results of our experiments show that EV-3 differs to EPO in terms of haematopoiesis but has functional similarities with regard to neuroprotection and cognitive enhancement. The mode of action of EV-3 is yet unknown but obviously independent of erythropoiesis. EPO is a promising candidate for the treatment of degenerative disorders^78^. A prominent and repeatedly reported observation for EPO in clinical studies is the beneficial effect of EPO on cognitive function^79–83^. Improvement of cognitive performance by EPO or its non-erythropoietic derivative carbamoylated EPO (CEPO) in mice^65, 66^ was partly explained by enhanced neurogenesis in the dentate gyrus^65^ and more recently by EPO-induced differentiation of precursors to neurons^45^. The literature provides evidence allowing refraining from the idea that EPO’s effects on memory function are merely mediated through haematopoietic action. The finding that EV-3 conserves EPO’s neuroprotective and cognition-promoting effects without stimulating erythropoiesis further supports the independency of these effects from erythropoiesis. Our study is limited by the number of EV-3 dosing regimens applied in the learning paradigm but provides novel and adequate results encouraging future studies with EV-3 to evaluate the potential of becoming a therapeutic for degenerative disorders. Simultaneously, the question regarding the mechanism of action of EV-3 arises. The binding site which mediates neuroprotective or cognitive effects of EV-3 is yet unknown and will be focus of future studies.

Taken together, our results provide evidence for EV-3 as the first example of a natural non-erythropoietic EPO protein isoform in humans. The observed co-regulation of *EPO* and its splicing variant hEPOΔ3 in human tissue together with the determination of the matching protein EV-3 in serum of healthy humans indicates the biological potential of our findings. Concomitant up-regulation of *EPO* and hEPOΔ3 under pathological conditions support our hypothesis of the involvement of both proteins in a tissue protective system and is exemplified by the neuroprotective effects against hypoxia-induced cell death of EPO and EV-3.

## Electronic supplementary material

Supplementary Information
