# Supplementary material for: EV-3, an endogenous human erythropoietin isoform with distinct functional relevance
Source: Sci Rep. 2017 Jun 16;7:3684. doi: 10.1038/s41598-017-03167-0 (PMC5473850; doi:10.1038/s41598-017-03167-0)
Supplement: Supplementary file 1 — Supplementary Information [file 41598_2017_3167_MOESM1_ESM.pdf]

*Supplementary Information*

# EV-3, an endogenous human erythropoietin isoform with distinct functional relevance

Christel Bonnas, Liane Wüstefeld, Daniela Winkler, Romy Kronstein-Wiedemann, Ekrem Dere, Katja Specht, Melanie Boxberg, Torsten Tonn, Hannelore Ehrenreich, Herbert Stadler, Inge Sillaber

## Supplementary methods

## Pharmacokinetic of EPO and EV-3 in mice

Male DBA/2J (Charles River) mice with an age of 8 weeks received a single i.p. injection of 5000 U/kg of recombinant EPO or EV-3 (IBA GmbH) and blood was sampled by decapitation following 1, 2, 4, 8, or 24 hours after injection (n=2 per time point and treatment). Following centrifugation (20 min, 3500 rpm, 4°C), the individual sera were frozen and stored (-80°C). EV-3 and EPO levels were determined by Sandwich-ELISA using StrepMAB-Immo-antibody (IBA GmbH) as capture antibody, to which the recombinant EV-3 or EPO in the serum samples bind via their Strep-tag. The polyclonal anti-EPO antibody H-162 (Santa Cruz) was used as detection antibody as the antibody binds both to EPO and EV-3. The plates (96 well, Maxisorb Nunc 442404, Thermo Scientific) were incubated overnight at 4°C with the capture antibody StrepMAB-Immo (50 µg/well), washed 3-times with PBS containing 0.05% Tween-20 (washing solution, 300 µl/well) and incubated with blocking solution (3% BSA in PBS, Sigma) for 90 min at RT on a shaker. Following a single wash, 50 µl of each serum sample was applied to the wells in duplicate and incubated for 150 min at RT. Serum samples from untreated mice were spiked with different concentrations of recombinant EV-3 or EPO and served as standards. After incubation of the samples, the wells were washed 3-times with washing solution and incubated with 50 µl of the diluted detection antibody H-162 (dilution 1:200 in 0.3% BSA-PBS, final concentration of the antibody 1 µg/mL). Following 3-times washing, 50 µl of the anti-rabbit poly HRP antibody (Thermo Fisher Scientific, dilution 1:2000 in 0.3% BSA) were added to each well and incubated for 50 min at RT. After the final wash (3-times), 50 µl of the substrate TMB (Sigma T4444) were added to each well and staining was stopped by adding 200 µl of 2M sulfuric acid. OD-values at 450 nm and 590 nm were determined by means of FluoStar Optima (BMG Labtech), sample levels were automatically calculated on the basis of the standards.

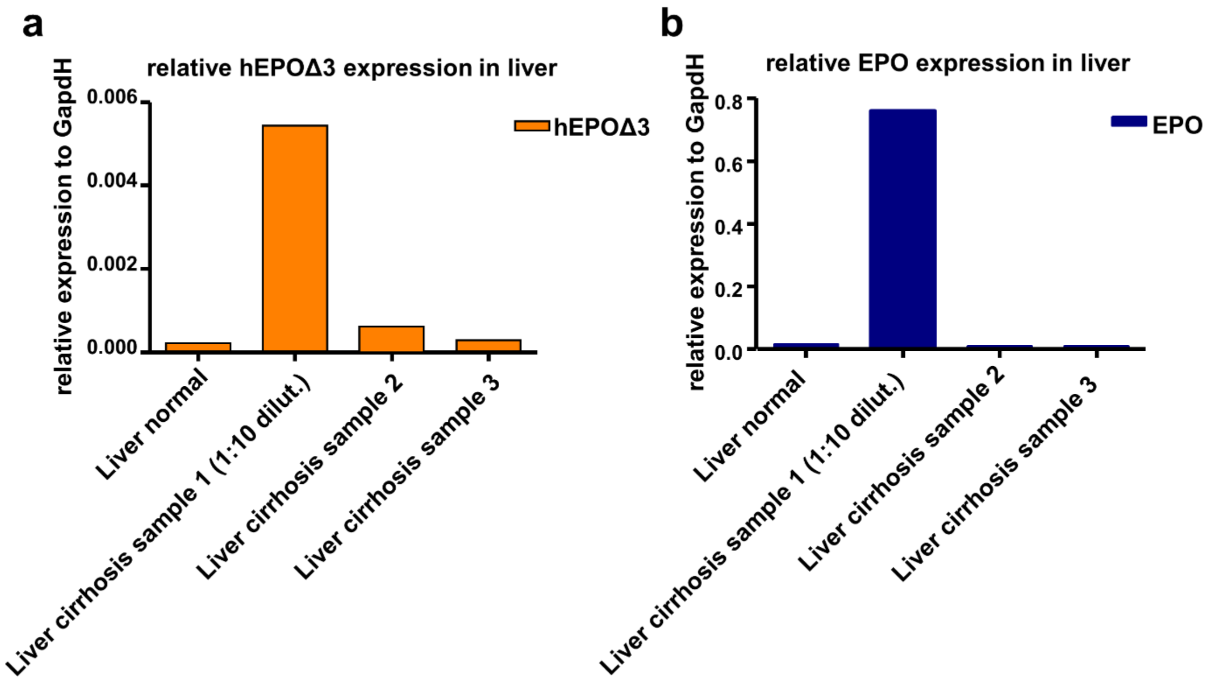


**Figure S1.** ***Expression analysis of EPO and hEPOΔ3 transcripts in normal and cirrhotic liver samples***

Relative *EPO* and hEPOΔ3 expression normalized to GapdH in a cDNA panel derived from commercially available human RNA samples from normal and cirrhotic livers hints to enhanced hEPOΔ3 (a) and *EPO* (b) expression in diseased condition (1/3 liver cirrhosis samples).

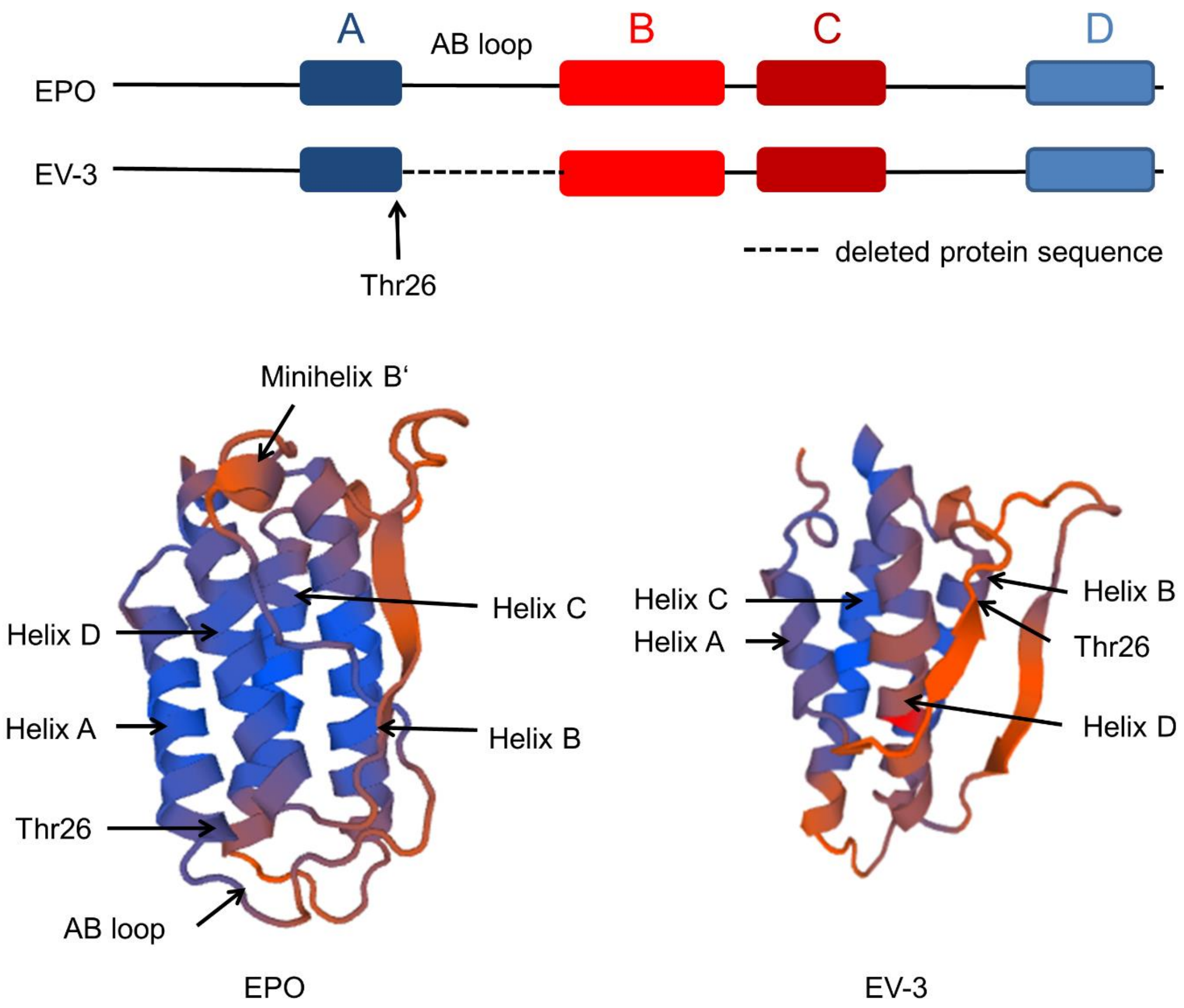


**Figure S2.** ***Protein structure prediction / modelling of EPO and EV-3***

EPO has a four-helical-bundle structure (helices A, B, C and D). In the AB loop is a short alpha-helical structure (minihelix B'). Loss of the region encoded by exon 3 in EV-3 is predicted to result in the absence of the AB loop. Structure prediction of EV-3 using an automated comparative protein modelling approach based on the EPO model 1cn4.1.C.pdb shows major changes in tertiary structure.

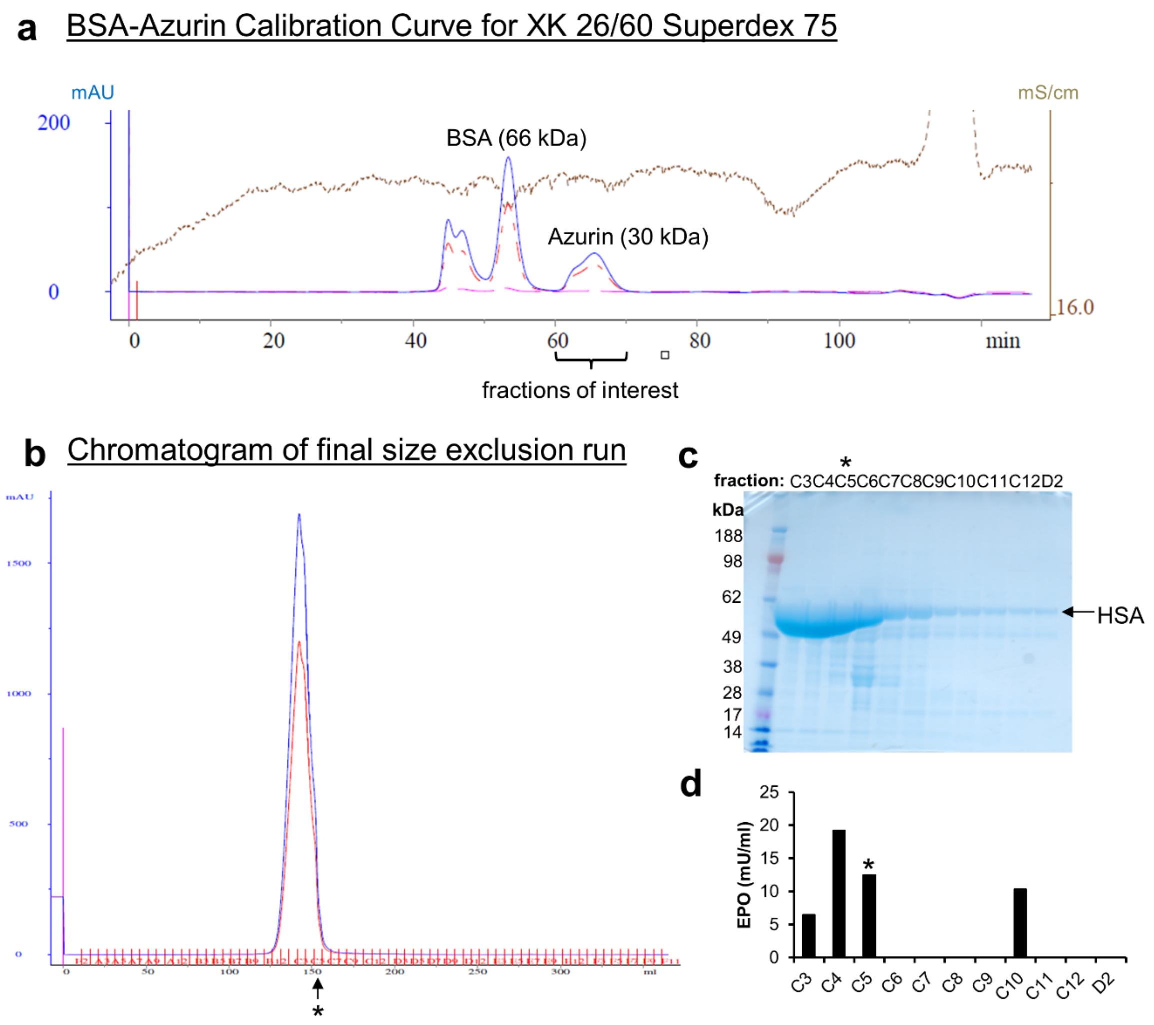


**Figure S3.** ***Size fractionation of serum 1 (20 ml) on a XK 26/60 Superdex column***

(a) Calibration run on the XK 26/60 Superdex column showing the fraction elution profile of Azurin from a BSA-Azurin solution. Positive fractions elute at time points 60-70 minutes (= fractions of interest). Azurin has a comparable molecular weight to EPO. Human serum fractions eluting at time points identified as fractions of interest from the calibration run were pooled, concentrated and applied to re-chromatography. Signal intensities are represented as dashed red line for UV absorbance at 260 nm, as solid blue line for UV absorbance at 280 nm and as broken pink line for UV absorbance at 310 nm. Conductivity of the buffer is shown as dotted brown line. (b) Chromatogram of the last size exclusion chromatography run on serum 1 showing a single narrow peak for serum albumin (B12-C6), indicating good separation from proteins smaller than albumin (fractions of interest are C4-C9 corresponding to elution time points 60–70 min). (c) The Coomassie gel shows the human serum albumin (HSA) content in the concentrated elution fractions (10x–20x concentrated) from the last size exclusion chromatography run on serum 1. Fractions C3-C6 show significant albumin content as indicated by the chromatogram from the final size exclusion run. (d) Using a commercial ELISA, EPO is detected in fractions C3-C5 (10x–20x concentrated) of serum 1.

* = fraction C5, which is further processed for LC-ESI-MS/MS.

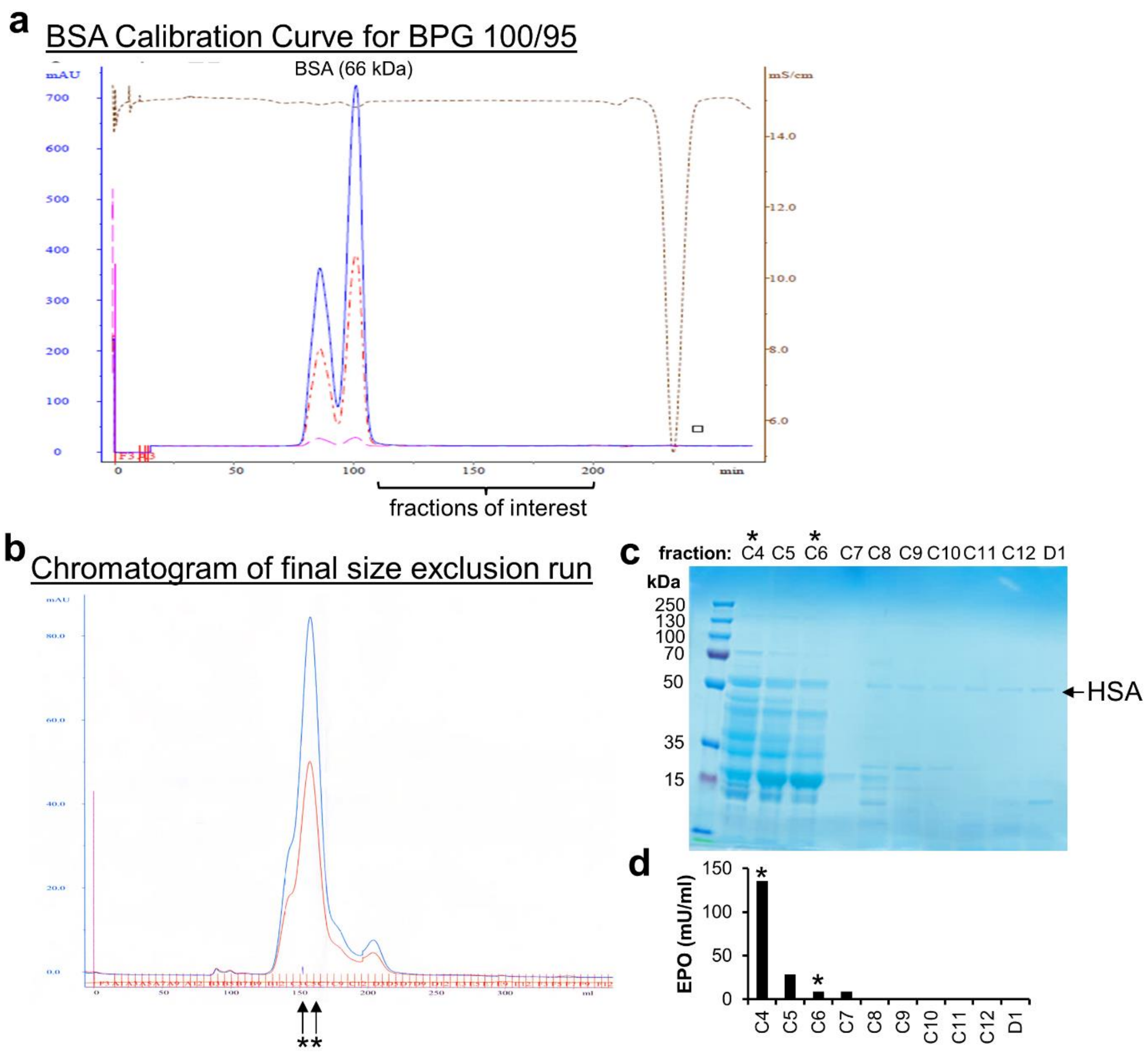


**Figure S4.** ***Size fractionation of serum 2 (100 ml) on a BPG 100/95 Superdex column and re-chromatography on a XK 26/60 Superdex column***

(a) Calibration run on the BPG 100/95 Superdex column showing the fraction elution profile of BSA. The fraction elution profile from EPO was estimated thereof (fractions of interest elute immediately after BSA). Human serum fractions eluting at time points identified as fractions of interest from the calibration run were pooled, concentrated and applied to re-chromatography. (b) Chromatogram of the last size exclusion chromatography run on serum 2 showing efficient albumin depletion (elutes in fractions B12-C6) and enrichment of proteins smaller than albumin (C6-D5). (c) The Coomassie gel confirms efficient human serum albumin (HSA) removal in the concentrated elution fractions (10x–20x concentrated) of serum 2. (d) Using a commercial ELISA, EPO is detected in fractions C4-C7 (10x–20x concentrated) of serum 2.

* = fractions C4 and C6, which are further processed for LC-ESI-MS/MS.

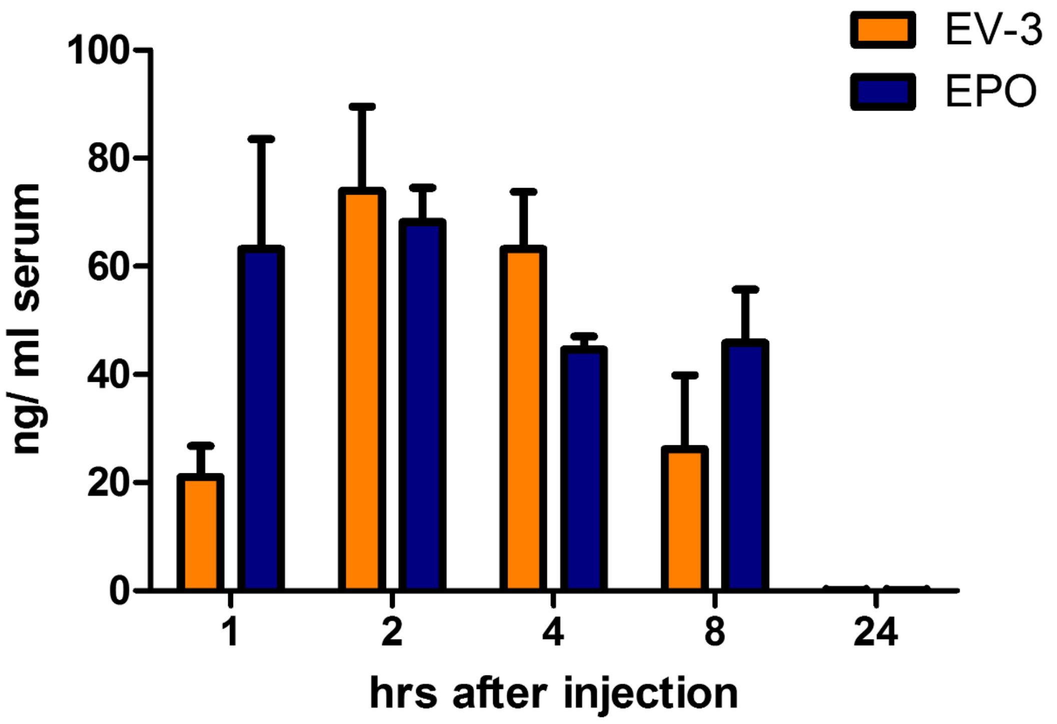


**Figure S5.** ***EV-3 and EPO levels in serum samples of mice after single i.p. injection***

Levels of strep-tagged EV-3 and EPO were determined by ELISA in serum collected of male DBA/2J mice at different time points (1, 2, 4, 8, or 24 hours; n=2 per time and treatment) after single i.p. injection of 42 µg/kg (approx. equivalent to 5000 U/kg) of recombinant EV-3 or EPO (IBA GmbH).
